# Posterior Reversible Encephalopathy Syndrome (PRES) After Acute Pancreatitis

**DOI:** 10.5811/westjem.2015.8.28347

**Published:** 2015-11-12

**Authors:** Tara Murphy, Khalid Al-Sharief, Vineeta Sethi, Gurpreet S. Ranger

**Affiliations:** * Dalhousie Medical School, Nova Scotia, Canada; † Upper River Valley Hospital, Waterville, New Brunswick, Canada

## Abstract

Posterior reversible encephalopathy syndrome (PRES) is an unusual condition typified by acute visual impairment caused by sudden, marked parieto-occipital vasogenic edema. Thought to be inflammatory in origin, it has been described in patients undergoing chemotherapy, with autoimmune disease, and in some infections. We report a case of PRES that occurred one week after an episode of acute pancreatitis in an otherwise healthy 40-year-old female. There was progressive visual impairment over a 24-hour period with almost complete visual loss, with characteristic findings on magnetic resonance imaging. After treatment with steroids, the visual loss recovered. Clinicians should retain an index of suspicion of this rare condition in patients with visual impairment after acute pancreatitis.

## INTRODUCTION

**Figure 1:**
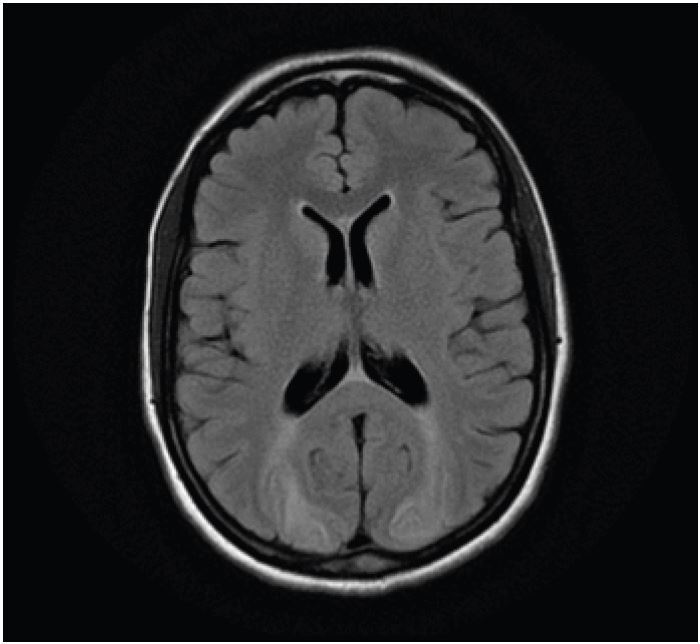
Magnetic resonance imaging scan showing symmetrical areas of increased signal in the occipital lobes (T2 and FLAIR sequences). *FLAIR*, fluid-attenuated inversion recovery

**Figure 2:**
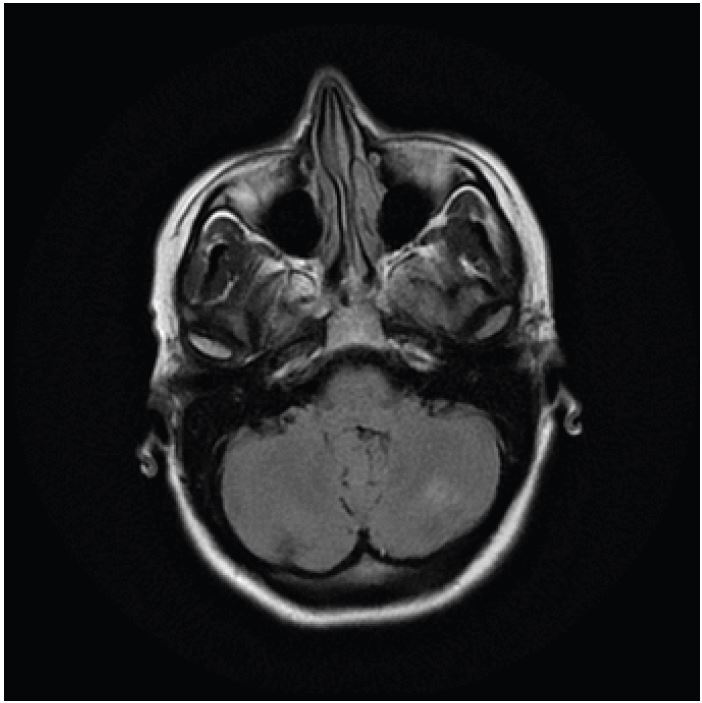
Magnetic resonance imaging scan showing wedge-shaped area of increased signal in the left cerebellar hemisphere (T2 and FLAIR sequences). *FLAIR*, fluid-attenuated inversion recovery

A 40-year-old female presented to our emergency department with sudden visual loss over a 24-hour period. She was otherwise healthy, but had been admitted two weeks previously with an episode of acute pancreatitis secondary to alcohol intake from which she had recovered uneventfully, and without any obvious sequelae. An urgent magnetic resonance imaging (MRI) scan was performed. This revealed symmetrical areas of hypoattenuation in both posterior parieto-occipital and cerebellar regions (Figures 1, 2). She was seen by a neurologist and diagnosed with posterior reversible encephalopathy syndrome (PRES). After a two-month course of steroids she had almost complete resolution of her vision and the radiological changes had improved.

## DISCUSSION

PRES is extremely rare, and usually diagnosed by a history of sudden visual impairment in the presence of specific radiological changes on MRI. Bilateral symmetrical hypodensitities in the parieto-occipital areas and cerebellar hemispheres on imaging are characteristic. The condition has been associated with chemotherapy, hypertension, infection and autoimmune disease.^1^

It is thought to occur from temporary impairment of the blood brain barrier causing vasogenic edema with symptoms of reduced consciousness, seizures, headaches, and typically visual problems.^2^ Around 26–67% of patients with PRES present with visual symptoms of blurred vision, visual neglect, homonymous hemianopsia, hallucinations or cortical blindness.

Our case is unusual, as PRES caused by pancreatitis has only been reported in very sick patients with other comorbidities. It probably occurred in this case as a result of the systemic inflammatory response.^3^,^4^,^5^,^6^

Whilst pancreatitis itself can be life threatening, this case reminds clinicians of unusual complications that can occur after discharge of patients who seem to have recovered from the disease.
